# Supplementary material for: Differential In Vitro Effects of SGLT2 Inhibitors on Mitochondrial Oxidative Phosphorylation, Glucose Uptake and Cell Metabolism
Source: Int J Mol Sci. 2022 Jul 19;23(14):7966. doi: 10.3390/ijms23147966 (PMC9319636; doi:10.3390/ijms23147966)
Supplement: Supplementary file 1 [file ijms-23-07966-s001.zip › ijms-1714915-supplementary.pdf]

International Journal of *Molecular Sciences*

MDPI

Structural features and physical properties of the SGLT-2 inhibitors

Chemical structures and physical properties of the four selective SGLT2 inhibitors are presented in Figure S1 and Table S1. Cana and ertu are more lipophilic than dapa and empa. Cana has a cLogP (pH 7.4) of 4.6, which is at least 28% higher than that of the other three SGLT2 inhibitors, a lower PSA (99) than the other SGLT2 inhibitors and a high plasma protein binding plasma protein binding (PPB: 99%).

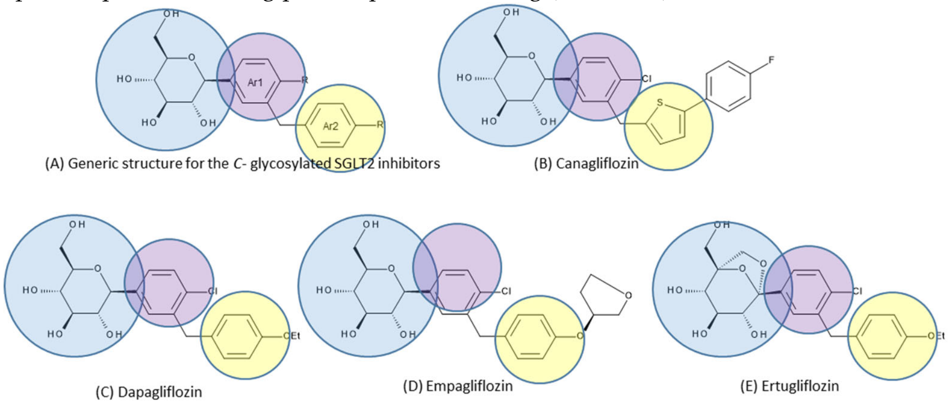

Figure S1: Chemical structures of SGLT2- inhibitors. Generic chemical structure of C-glycosylated SGLT2 inhibitors (A); of cana (B); dapa (C); empa (D); ertu (E). Ortho-substitution of a second aryl group is highlighted in yellow; arene is highlighted in purple; sugar moiety is highlighted in blue.

Table S1: Physical properties of SGLT2- inhibitors.

SGLT2 inhibitor	cLogD	cLogP (pH 7.4)	PSA	PPB
cana	3.7	4.6	99	99%
dapa	2.5	3.3	108	91%
ertu	3.9	3.1	117	94%
empa	2.4	2.6	116	86%
